# Population dynamics of *Agriophyllum squarrosum*, a pioneer annual plant endemic to mobile sand dunes, in response to global climate change

**DOI:** 10.1038/srep26613

**Published:** 2016-05-23

**Authors:** Chaoju Qian, Hengxia Yin, Yong Shi, Jiecai Zhao, Chengliang Yin, Wanyin Luo, Zhibao Dong, Guoxiong Chen, Xia Yan, Xiao-Ru Wang, Xiao-Fei Ma

**Affiliations:** 1 Key Laboratory of Stress Physiology and Ecology in Cold and Arid Regions, Gansu Province, Department of Ecology and Agriculture Research, Cold and Arid Regions Environmental and Engineering Research Institute, Chinese Academy of Sciences, Lanzhou 730000, Gansu, China; 2 University of Chinese Academy of Sciences, Beijing 100049, China; 3 Key Laboratory of Desert and Desertification, Cold and Arid Regions Environmental and Engineering Research Institute, Chinese Academy of Sciences, Lanzhou 730000, Gansu, China; 4 Key Laboratory of Eco-hydrology and of Inland River Basin, Cold and Arid Regions Environmental and Engineering Research Institute, Chinese Academy of Sciences, Lanzhou 730000, Gansu, China; 5 Department of Ecology and Environmental Science, Umeå University, Umeå 90187, Sweden

## Abstract

Climate change plays an important role in the transition of ecosystems. Stratigraphic investigations have suggested that the Asian interior experienced frequent transitions between grassland and desert ecosystems as a consequence of global climate change. Using maternally and bi-parentally inherited markers, we investigated the population dynamics of *Agriophyllum squarrosum* (Chenopodiaceae), an annual pioneer plant endemic to mobile sand dunes. Phylogeographic analysis revealed that *A. squarrosum* could originate from Gurbantunggut desert since ~1.6 Ma, and subsequently underwent three waves of colonisation into other deserts and sandy lands corresponding to several glaciations. The rapid population expansion and distribution range shifts of *A. squarrosum* from monsoonal climate zones suggested that the development of the monsoonal climate significantly enhanced the population growth and gene flow of *A. squarrosum*. These data also suggested that desertification of the fragile grassland ecosystems in the Qinghai-Tibetan Plateau was more ancient than previously suggested and will be aggravated under global warming in the future. This study provides new molecular phylogeographic insights into how pioneer annual plant species in desert ecosystems respond to global climate change, and facilitates evaluation of the ecological potential and genetic resources of future crops for non-arable dry lands to mitigate climate change.

Climate change plays an important role in the transition of ecosystems^1^,^2^,^3^. Compared with other ecosystems, desert ecosystems might be much more sensitive to climate change because these environments feature low biodiversity and high environmental stress^4^,^5^. One of the most striking results of climate change in the Northern Hemisphere during the Cenozoic era is the desertification of the Asian interior, which can be traced to ~22 Ma^6^. In the Quaternary period, temperate deserts experienced large-scale expansions in marine isotope stage 16 (MIS 16, ~0.6–0.7 Ma) and in the last glacial maximum (LGM, ~21–18 Ka)^7^. During MIS 16, the maximum global ice volume and successive Kunhuang Movement induced the intensification of the East Asian Winter Monsoon (EAWM), thus resulting in the substantial expansion of the deserts in marginal monsoonal regions^7^,^8^. In the LGM period, the dominant EAWM induced the expansion of deserts in marginal monsoonal zones compared with the deserts in non-monsoonal regions^7^,^9^. However, in the warmer period of the Holocene Optimum (HO, ~9–5 Ka), when the East Asian Summer Monsoon (EASM) dominated, due to the reduced wind velocity and increased precipitation, all the sandy lands were completely stabilised, and their desert ecosystems fully transitioned into grassland ecosystems, by contrast, deserts far from the monsoonal zone contracted only 5% to 20%, as compared with their present ranges^9^. These phenomena indicate that monsoonal climate oscillations have frequently affected the vegetation transition of desert ecosystems and the temporal-spatial evolution of deserts in the Asian interior.

Although perennial plants have a high tolerance to climate change^10^, our new molecular phylogeographic data have demonstrated that the East Asian monsoon system (EAMS) could have significantly affected intraspecific divergence, gene flow and regional population dynamics of *Reaumuria soongarica*, a constructive shrub of temperate desert ecosystems^11^. However, we did not detect a significant relationship between the demographic history of this perennial desert plant and the spatial-temporal evolution of these temperate deserts. Because the demographic history of annual plants from diverse ecosystems should be more sensitive to environmental changes^12^,^13^,^14^, the population dynamics of the annual plants from the desert ecosystem might respond more rapidly to changes in the monsoonal climate. Therefore, a thorough demographic investigation and distribution range reconstruction and prediction for annual plants may provide an ideal platform to fully understand how desert ecosystems have responded to climate change in the past and to predict how these environments will respond in the future.

*Agriophyllum squarrosum*, also called “sand rice”, is a typical annual pioneer desert plant of the Chenopodiaceae family^15^,^16^. Among its sister species, only *A. squarrosum* is widely distributed in the mobile sand dunes of all deserts and sandy lands across the Asian interior (http://foc.eflora.cn) and can survive extremely high temperatures and drought and tolerate sand burial^17^,^18^,^19^. Because the withered plants reduce wind velocity by at least 90% and are a rich source of carbon and nitrogen in these poor soil environments, *A. squarrosum* plays a critical role in sustaining and restoring fragile desert ecosystems^20^,^21^. Furthermore, although this plant grows in infertile and sandy soils, *A. squarrosum* has a high concentration of nutrients in its seeds and a high biomass, thus making this plant an invaluable candidate species for domestication as a food and forage crop for dry lands^22^, More interestingly, the colonisation of *A. squarrosum* is often accompanied by the shifting of sand dunes, thus providing an ideal model to understand the historical dynamics of desert ecosystems in response to climate change.

In the present study, we investigated the distribution of species-wide standing genetic variation in *A. squarrosum* by using two marker systems: five maternally inherited chloroplast DNA (cpDNA) fragments^23^ and a bi-parentally inherited nuclear ribosomal internal transcribed spacer (nrITS) region. By reconstruction of genealogical networks, profiling of population dynamics and prediction of shifts in the distribution range of this annual pioneer desert plant, we sought to address the following questions: i) whether the colonisation of *A. squarrosum* is associated with palaeo-climate changes; ii) whether and how the monsoonal climate affects the genetic differentiation, gene flow and population dynamics of *A. squarrosum*, as the habitat of this plant is characterised by mosaic deserts and the high mobility of sand dunes, both of which are frequently affected by monsoonal climate change; and iii) how the distribution range of this annual pioneer plant has shifted and will shift in the future in response to global climate change. From a molecular phylogeographic perspective, this endeavour will shed light on the current understanding of how desert ecosystems respond to changes in monsoonal climates, thus facilitating the evaluation of the ecological potential and germplasm resources of breeding candidate crops to manage the food supply in harsh environments.

## Results

### Geographical structure of ribotypes and chlorotypes

The nrITS and five cpDNA fragments of a total of 188 ingroup individuals from 46 populations and 7 outgroup individuals were sequenced. A total of 586 bp of nrITS alignment and 35 ribotypes were obtained, which were defined by 29 polymorphic sites, including one indel (Supplementary Table S1). Based on the concatenated chloroplast supergene of 3,401 bp, we identified 13 chlorotypes, defined by 18 polymorphic sites, including 2 indels (Supplementary Table S2). The overall average haplotype diversity (*Hd*) of *A. squarrosum* populations was 0.691 on nrITS and 0.646 on cpDNA, respectively. The average overall nucleotide diversity (*pi*) was 0.0025 on nrITS and much lower on cpDNA (*pi* = 0.0004) (Supplementary Table S1 and S2), consistently with the selfing of this annual plant, thus confirming the reliability of this sampling strategy. Among all populations, population BEJX from group GuD (for detailed information about this group, refer to Methods) harboured the highest genetic diversity (*pi* = 0.0044 on nrITS and 0.0014 on cpDNA, respectively).

**Figure 1:**
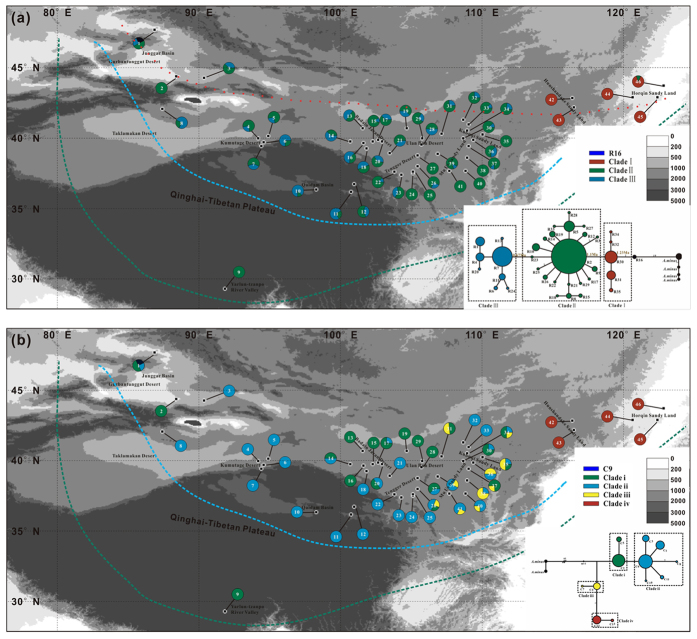
The distribution of haplotypes in the geological locations and Median-joining networks of the haplotypes in the 46 *A. squarrosum* populations examined in this study. (**a**) nrITS ribotype distribution and genealogy topology; (**b**) cpDNA chlorotype distribution and genealogy topology. Population locations are presented as small black spots on the map, and the slice size is proportional to the frequency of the haplotypes (different clades). The colours of the pie-diagrams correspond to clades of the network genealogies at the bottom of the two maps. The sizes of the circles in the genealogy topology correspond to the frequency of each haplotype; each number near the line represents the mutational steps interconnecting two haplotypes, and only the steps over two mutations are listed. The red dotted line represents the first wave of colonisation, the green dashed line represents the second wave of colonisation, and the blue dashed line represents the third wave of colonisation. These figures originated from the software packages of Diva-GIS version 7.5.0 (http://www.diva-gis.org/), and then modified by Q,C.J. with CorelDraw X6 (Corel Corporation, Ottawa, Canada).

The genealogies and frequencies of the 35 ribotypes and 13 chlorotypes were projected onto a geographical map (Fig. 1a,b, respectively). The ancestral ribotype of R16, which directly connected with the outgroup ribotypes, was restricted to population BEJX from the GuD group. The other ribotypes can be distinctly separated into the following clades: clade I, clade II and clade III, in which the ribotypes from clade I were fixed in the East group. The ribotypes from clade II covered all deserts and sandy lands, even reaching the southern edge of the QTP and eastern edge of the East sandy lands. In comparison, the area that the ribotypes from clade III covered was much smaller and was limited to the deserts from the western, central and northeastern QTP (Fig. 1a).

Similarly to the ribotypes, the ancestral chlorotype C9 was also restricted to the population BEJX. The main chlorotypes were separated into 4 clades: clade i, clade ii, clade iii and clade iv (Fig. 1b). Interestingly, the geographical distributions of the chlorotypes from clades i and ii were similar to those of the ribotypes from clades II and III, respectively. The genealogical topologies and geographic distribution patterns of the two markers strongly suggested that *A. squarrosum* originated from the Gurbantunggut desert and subsequently experienced at least two waves of colonisation into other deserts. This pattern of the origination and dispersal of *A. squarrosum* was well supported by the data obtained from the RASP analysis (Supplementary Fig. S1).

### Time estimation of the divergence and diversification of lineages

**Figure 2:**
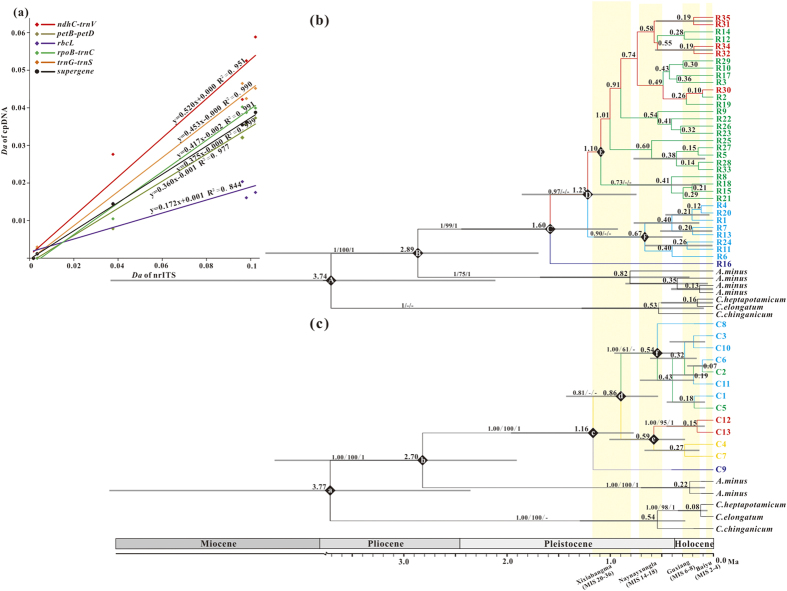
Beast-generated maximum clade credibility tree of 35 ribotypes (R1-R35) and 13 chlorotypes identified in *A. squarrosum* and haplotypes in outgroups. (**a**) The correlation of divergence between different species for the nrITS, five chloroplast fragments and the supergene separately: x-axis, *Da* of nrITS; y-axis, *Da* of chloroplast fragments. Different colours represent different chloroplast fragments; (**b**) BEAST tree estimated using the ribotypes; (**c**) BEAST tree estimated using the chlorotypes. The numbers adjacent to the nodes indicate bootstrap percentages from BEAST, ML and BI. The symbol - indicates that the analysis does not support this branch. The node age (Ma) estimates are marked by letters; a-f represents the coalescence time of each major clade, and the length of the grey bars represents the 95% highest posterior density.

Based on the calibrated mutation rates of the nrITS and chloroplast supergene, BEAST analysis indicated that the genera *Corispermum* and *Agriophyllum* might have diverged at ~3.74 Ma (95% HPD, 2.1 – 5.9 Ma) based on nrITS and ~3.77 Ma (95% HPD, 1.9 – 7.2 Ma) based on cpDNA, respectively (points “A” and “a” in Fig. 2b,c), thus suggesting that this calibration was objective. The two species of genus *Agriophyllum* might have diverged at ~2.7 Ma based on cpDNA (95% HPD, 1.3–4.8 Ma) and ~2.9 Ma based on nrITS (95% HPD, 1.7–4.4 Ma; point “b” and “B” in Fig. 2b,c), associated with the formation of the modern Asian monsoon and the rapid growth of glaciers in the Pliocene^24^,^25^. The age of the crown nrITS lineages of *A. squarrosum* could be dated to ~1.6 Ma; however, the ages of the crown main lineages of the chlorotypes were much later than the ribotypes, which did not begin diversifying until ~1.16 Ma (points “C” and “c” in Fig. 2b,c). Consequently, the ages of the crowns of the three main clades of ribotypes were dated to ~1.23 Ma, ~1.10 Ma and ~0.67 Ma, respectively (points “D”, “E” and “F” in Fig. 2b).

### Population differentiation and gene flow

The PERMUT results for nrITS revealed that the total genetic diversity of all populations was much higher than the average diversity within populations (*H*_T_ = 0.750 and *H*_S_ = 0.569, respectively), consistently with the significant phylogeographic structure (*Nst* > *Gst*, *p*-values = 0.019, Table 1). The hierarchical molecular variation analysis (AMOVA) also supported the significant phylogeographic structure of *A. squarrosum*, as the genetic differentiation among the five groups was higher than that among the populations within groups (*F*_CT_ = 0.288, *F*_SC_ = 0.154, *F*_ST_ = 0.397, Table 2). Although a significant phylogeographic structure could not be detected on maternal inherited cpDNA (Table 1), the pairwise genetic differentiation was significantly high between the East and other groups (Table 3, Supplementary Fig. S2). However, we observed the lowest *F*_ST_ between the QTP group and the Central group but the highest *F*_ST_ among populations within the QTP group on cpDNA (0.133 and 1.000, respectively).

**Figure 3:**
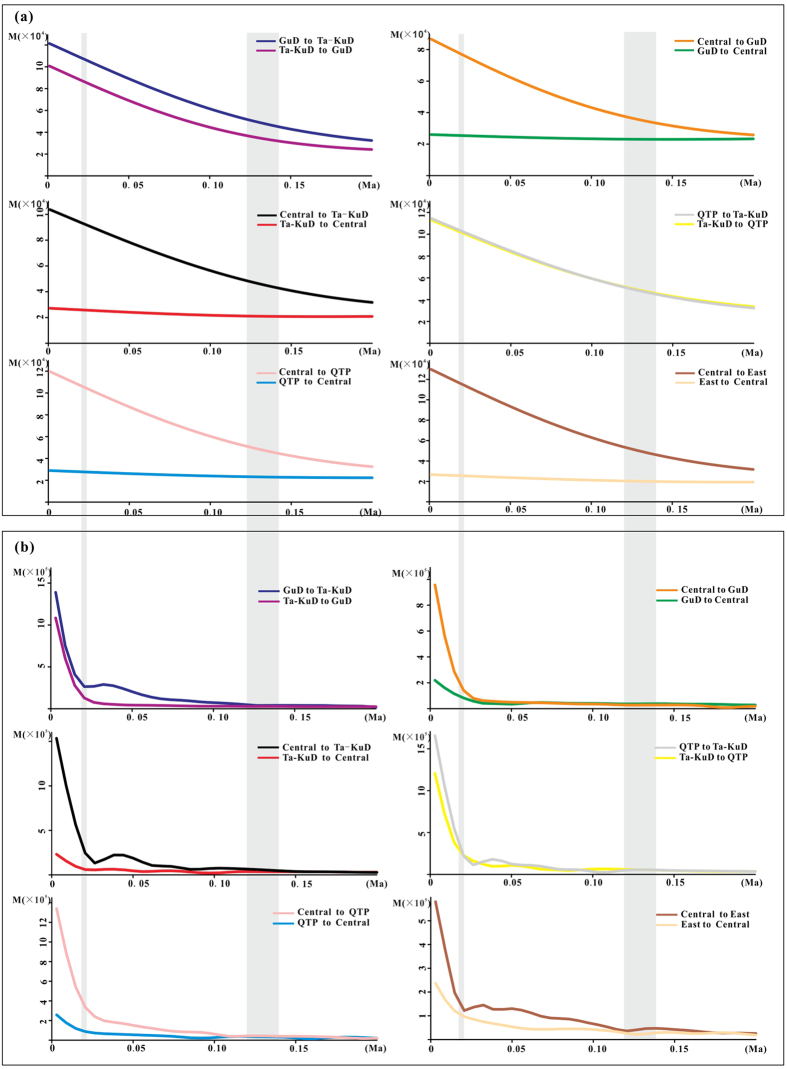
Gene flow estimate by Migrate-n for each group of *A. squarrosum*. (**a**) Gene flow estimated using nrITS data, (**b**) gene flow estimated using cpDNA data.

When the East group was included, the results of the Mantel test revealed significant correlations between the genetic differentiation and physical distance on both nrITS and cpDNA (r = 0.489, *p* < 0.0001; r = 0.3077, *p* < 0.0001, respectively). However, we did not detect the signals when the East group was excluded (r = 0.187, *p* = 0.0258 for nrITS; r = 0.069, *p* = 0.1748 for cpDNA, respectively), indicating the occurrence of frequent gene flow among the groups, except the East group. However, the gene flow among the groups was markedly unbalanced, because the Migrate-n analysis demonstrated that the gene flows from the Central group were much larger than those in other directions (Fig. 3). We also observed that the maternal gene flow was significantly enhanced during the LGM, which comprehensively merged the genetic differentiation among the groups, except the East group (Fig. 3b).

### Demographic history of *A. squarrosum*

**Figure 4:**
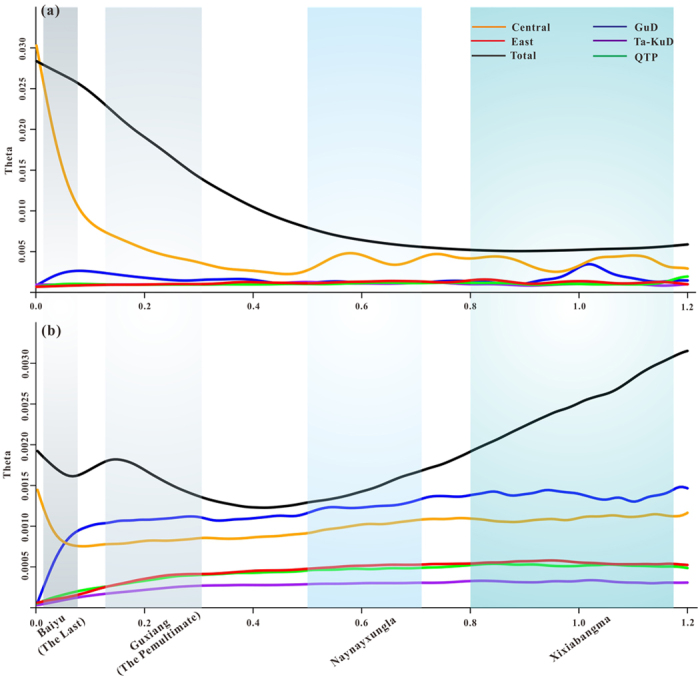
Skyline plot estimated by Migrate-n for each group of *A*. *squarrosum*: x-axis, time in million years ago; y-axis, theta (θ = 4 μNe for nrITS; θ = 2 μNe for cpDNA). Letters after horizontal lines with different colours represent the skyline plots for the five groups and the whole populations, respectively.

The significantly negative values of Tajima’s *D* (−1.801, *p* = 0.008) and Fu’s *Fs* (−27.231, *p* = 0.000) for the nrITS data reflect a significant expansion of the overall populations of *A. squarrosum* (Table 4). Skyline plots of nrITS also demonstrated that the overall population size of *A. squarrosum* significantly increased since ~0.8 Ma (Fig. 4a). Although neutrality tests did not support the significant expansion of the overall populations on cpDNA (Table 4), skyline plots demonstrated that the total population size experienced an expansion since ~0.4 Ma, a decline from 0.15–0.07 Ma and another rapid expansion during the LGM (Fig. 4b), indicating that the effective population size of the maternal inherited cpDNA of this pioneer annual desert plant was highly sensitive to changes in the monsoonal climate. Among the different geographical groups, only the Central group experienced dramatic population growth since ~0.42 Ma based on nrITS and since the LGM, based on cpDNA (Fig. 4), and the growth of this group was largely responsible for the overall population growth of *A. squarrosum*. However, the GuD group experienced a significant bottleneck in both nrITS and cpDNA during the LGM (Fig. 4).

### Distribution range shift under climate scenarios

**Figure 5:**
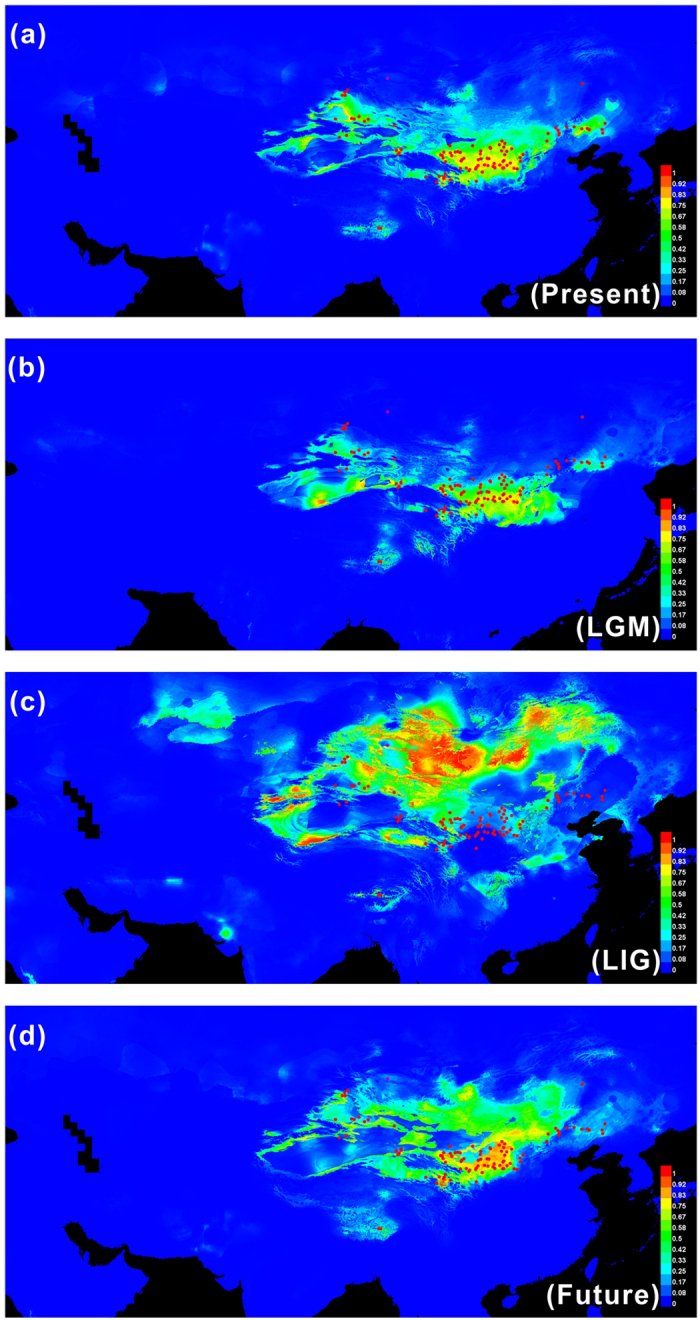
Predicted distributions of *A. squarrosum*. The red plots represent the 124 localities, including our own collection sites, and records from the Chinese Virtual Herbarium were used to construct the models. These figures originated from the software packages of MAXENT version 3.3.3, the environmental variables originated from from the WorldClim database version 1.4 (1950–2000, http://www.worldclim.org/), and then modified by Q,C.J. with CorelDraw X6 (Corel Corporation, Ottawa, Canada).

The high AUC scores (AUC = 0.963 ± 0.051) of ENM supported the excellent goodness of our models, and the predicted niche range of *A. squarrosum* covered all deserts and sandy lands across the Asian interior (Fig. 5a). Because the distribution ranges of *A. squarrosum* were largely affected by precipitation and mean temperature of the coldest quarter (Supplementary Table S3), an extremely cold and dry climate was not suitable for this species. If the climatic niche of *A. squarrosum* was conserved throughout its evolutionary history, then the shift in the distribution range of this pioneer annual desert plant might reflect how the desert ecosystem responded to climate change. The simulations revealed that during the cooling of the LGM, the distribution range of *A. squarrosum* fragmented and shifted into lower latitude regions, even occupying the entire Loess Plateau (Fig. 5b). In comparison, during the warming of the LIG, the suitable habitats might have extended to higher latitude regions and nearly covered all deserts across the Asian interior. We also observed that all sandy lands might have transitioned to grasslands, which would not have been suitable for *A. squarrosum* at that time (Fig. 5c). In a warmer scenario in the near future, we predict that the habitat of *A. squarrosum* will expand into higher latitude and higher altitude regions, such as the Mongolia Plateau and the QTP (Fig. 5d). The distribution patterns during the warmer climate in both the past and future strongly indicate that these high Asian regions may be at great risk of desertification under global warming.

## Discussion

The geographic patterns of the genetic diversity and distribution of species are comprehensively affected by past geological and climatic changes^26^. Here, we thoroughly investigated the phylogeographic structure, population dynamics and shifts in the distribution range of *A. squarrosum*, an annual pioneer plant endemic to mobile sand dunes, to infer the dynamic response of desert ecosystems to global climate change.

According to the calibrated mutation rates, the two *Agriophyllum* species may have diverged at ~2.89 Ma (on nrITS) and/or ~2.70 Ma (on cpDNA) (Fig. 2a,b, respectively), when the glaciers rapidly developed, the modern East Asian Monsoon formed and the Asian interior was significantly dried according to the sedimentology records^27^,^28^,^29^. According to the distribution analysis of ancestral haplotypes using RASP, *A. squarrosum* might have originated in the Gurbantunggut region (Fig. 1, Supplementary Fig. S1). Therefore, we suspect that the aridification process at that time generated the mobile sand dunes on a small scale (most probably in the Gurbantunggut region), thus creating a novel habitat for *A. squarrosum* and accelerating the divergence between *A. squarrosum* and *A. minus*. If we arbitrarily ignore the differentiation between the times of lineage diversification and dispersal, then the crown age of the corresponding lineages may be regarded as the time at which the ancestral ribotypes appeared. Thus, the first colonisation of *A. squarrosum*, limited in regions of high latitude, was not as early as the crown age of clade I (~1.23 Ma, Fig. 2b). However, considering the high mobility of sand dunes, the present distribution of the ribotypes from clade I might not fully reflect the original pattern. On the other hand, unfortunately, samples from minor scattered distribution regions, such as the Caucasus, Siberia and eastern Europe (http://www.ars-grin.gov/cgi-bin/npgs/html/taxon.pl?310926) were not included. Therefore, the range of the first wave of colonisation might have been underestimated in this analysis.

Although the time of lineage diversification of maternal inherited chlorotypes was substantially delayed compared with that of bi-parental inherited ribotypes, the geographical distribution of the chlorotypes was fairly similar to that of the ribotypes, strongly supporting the last two waves of the colonisation (Fig. 1b). The distribution ranges of the ribotypes from clade II and chlorotypes from clade iii suggest that the second wave of *A. squarrosum* colonisation might have spread to all deserts and sandy lands in the Asian interior, dated to ~1.1 Ma. This time of colonisation is consistent with Xixiabangma glaciation (~0.8–1.17 Ma), and during this period, the EAWM were significantly intensified and thus could have greatly induced the expansion of these temperate deserts^30^. Compared with the second wave of colonisation, the third wave occurred more locally and recently, as the crown age of clade III was dated to ~0.67 Ma (Fig. 2b, point “F”), consistently with the Naynayxungla Glacial Period (~0.5–0.72 Ma)^30^. Because the new habitat was a prerequisite for the colonisation of this pioneer desert plant, these three waves of colonisation strongly suggest that the temperature deserts of the Asian interior underwent at least two significant expansions as a consequence of the glacial cycles, and the global cooling significantly contributed to the stepwise drying of the Asian interior as suggested in previous studies^24^,^31^.

As shown in the skyline plots of the population size based on both nrITS and cpDNA data, the regional groups experienced diverse population dynamics. The Central group from the monsoonal climate zone underwent significant expansion as the primary contributor to the overall population growth of the species. Similarly, the population of another desert shrub, *R. soongarica*, from the monsoonal climate zone grew much faster than did other regional populations^11^. Because of the seesaw battles between summer and winter monsoons during Pleistocene climatic oscillations, populations from the monsoonal zone probably experienced frequent population bottlenecks and habitat fragmentation, which might have further promoted the fixation of rare alleles within the local populations^32^,^33^. In addition, the significant population expansion of the Central group might also have enhanced maternal and bi-parental gene flow from the Central group to the other groups rather than the opposite direction (Fig. 3). Notably, the maternal gene flow was dramatically enhanced during the LGM, indicating that the Central deserts, particularly the sandy lands, significantly expanded during the LGM. From a molecular phylogenetic perspective, this result strongly supports stratigraphic investigations of the desert regions of the Asian interior^9^.

Because the population structure and frequent gene flow can produce false signals of population decline^34^, extreme caution should be applied when analysing the apparent dramatic population decline in the GuD group (Fig. 4). We observed that both the maternal and bi-parental gene flows were much higher from the Central group to the other groups than in the opposite direction. However, the populations of the other groups did not decline as rapidly as those from the GuD group. In contrast, compared with other groups, the predicted suitable habitat in the Gurbantunggut region significantly decreased (Fig. 5), which might have induced a loss of genetic diversity. Therefore, although gene flow was frequent, the population of the GuD group might have truly declined as a result of habitat loss.

Although biotic interactions were documented as the main shortage in ecological niche modelling in most cases^35^, *A. squarrosum* is one of a few pioneer annual desert plants endemic to mobile sand dunes, biotic interactions might play a negligible role in shaping its distribution range. Therefore, modelling the shift in the distribution range of this plant under climate scenarios is instructive for elucidating the response of temperate desert ecosystems to climate change. In contrast with the cold, dry climate of the LGM period, both past and future global warming might result in the substantial expansion of the distribution range of *A. squarrosum* to high Asian regions, including the Mongolia Plateau, and the QTP, which suggests that the grassland ecosystems of high Asia will be at great risk of degradation to desert ecosystems with on-going future warming (0.6 degrees per century)^36^. Interestingly, another artificial warming experiment has also demonstrated that the grassland of the QTP tends to be degraded^37^, thus further supporting our simulation of the ecological niche model of *A. squarrosum*.

Interestingly, previous stratigraphic investigations on the loess deposits have demonstrated that the oldest loess deposits in the Qaidam Basin and Yarlun-Tzanpo River Valley can be traced to ~14.9 Ka and ~13.0 Ka, respectively^38^,^39^, suggesting that the desertification of the QTP was fairly recent. However, the data in the present study indicated that *A. squarrosum* may have invaded the QTP along with the second wave of colonisation at ~1.1 Ma. Other numerous sedimentology and aeolian records^8^,^28^,^40^ have also indicated that during that time, the QTP experienced several glaciations and the EAWM intensified, largely inducing the expansion of these temperature deserts, even into the southern edge of the QTP.

Furthermore, the occurrence of the maximum *F*_ST_ between the populations within the QTP group on both nrITS and cpDNA, also suggests that the two main regional populations (North-eastern QTP and Yarlun-Tzanpo River valley) of *A. squarrosum* were significantly differentiated, and the gene flow was effectively stopped by the QTP. Because the lowest *F*_ST_ based on cpDNA occurred between the Central and QTP groups, we concluded that maternal gene flow between the two groups was sufficiently large to break through the geological barrier of the Qilian Mountain but could not pass through the QTP. Therefore, the desert of the Yarlun-Tzanpo River Valley may have been formed before the rapid uplift of the QTP in mid-Pleistocene, which reached 3000 m to 4000 m^41^,^42^.

In conclusion, based on the detailed analysis of the population dynamics and shifts in the distribution range of the annual pioneer desert plant, this study provides new insights on how desert ecosystems respond to climate change. These data also indicated that the desertification of the QTP may have occurred as early as approximately 1.1 Ma. Interestingly, under global warming scenarios, the potential range of this species may expand into regions of high Asia, thus suggesting a great risk of desertification in these regions. The present study further contributes to the understanding of the evolutionary history of the plant communities in central Asian deserts and to the evaluation of the ecological potential of a future crop uniquely adapted to the harsh desert climate.

## Methods

### Sample collection

A total of 188 individuals from 46 natural populations of *A. squarrosum* were sampled across all deserts and sandy lands in the Asian interior (see Supplementary Table S4, Fig. 1). Three species from the genus *Corispermum* and four individuals of *A. minus* were collected as outgroups. Fresh leaves were dried and preserved in silica gel, and voucher specimens were deposited in the Key Laboratory of Stress Physiology and Ecology in Cold and Arid Regions, Cold and Arid Regions Environmental and Engineering Research Institute, Chinese Academy of Sciences.

### DNA extraction, PCR and sequencing

Total genomic DNA was extracted with Plant Genomic DNA Kits (Tiangen Biotech Co., LTD, Beijing, China). After the preliminary screening of 12 pairs of cpDNA fragments within 10 individuals from five long-distance populations, five cpDNA fragments (*ndhC/trnV*, *petB/petD*, *rbcL*, *rpoB/trnC* and *trnS1/trnG1*) and one nrITS fragment (*ITS1/ITS4*) were selected for the phylogeographic survey (Supplementary Table S5). PCR was performed using 2 X Taq Plus high fidelity PCR Master Mix (Tiangen, Beijing, China) in a Gene-Amp PCR system 9700 DNA Thermal Cycler (PE Applied Biosystems, Norwalk, USA) using the programmes listed in Table S5. The DNA products were purified using TIAN quick Midi Purification Kits (Tiangen, Beijing, China) and were subsequently sequenced with both forward and reverse primers on an ABI 3130xl Genetic Analyzer (PE Applied Biosystems, Norwalk, USA) using ABI Prism BigDye Terminator Cycle version 3.1 (PE Applied Biosystems, Norwalk, USA).

All newly obtained sequences were deposited in GenBank under accession numbers KT229649–KT229733.

### Estimation of genetic differentiation and population dynamics

All DNA sequences were verified with BioEdit version 7.1.3^43^ after alignment using MUSCLE (available online: http://www.ebi.ac.uk/Tools/msa/muscle/). A chloroplast supergene was concatenated from the five cpDNA fragments with DnaSP version 5.10.01^44^. The heterozygous sites of the nrITS sequences^45^ were separated with PHASE, which was integrated in DnaSP^46^.

SAMOVA was used to infer the geographic structure of both markers by identifying the number of population groups (K) with the highest *F*_CT_ value^47^. After re-sampling 100 datasets with POPTOOLS version 3.2.5^48^, 100 pairwise *F*_ST_ matrices were estimated with Arlequin version 3.11^49^, and the genetic barrier was detected with BARRIER version 2.2^50^. To verify the association between genetic isolation and geographical distance, Mantel tests^51^ with 10,000 randomisations were performed on both nrITS and cpDNA using the web service of IBDWS version 3.23 (http://ibdws.sdsu.edu/~ibdws/distances.html).

According to the results from SAMOVA, BARRIER and monsoonal climatic characteristics of their geological locations^9^,^52^,^53^, all populations were divided into the following 5 groups (see Supplementary Table S4 and Fig. S2): GuD, Ta-KuD, Central, QTP and East. Molecular diversity parameters were calculated for both markers using DnaSP (Supplementary Table S1 and S2), and the haplotype diversity of total-populations and within-populations (*h*_T_ and *h*_S_) and the ordered (*N*_ST_) and unordered population differentiation (*G*_ST_)^54^ were calculated using PERMUT Version 2.0^55^ with 5,000 random permutations. The detailed genetic differentiation among groups (*F*_CT_), populations within groups (*F*_SC_) and within populations (*F*_ST_) were estimated using AMOVA implemented in Arlequin with default settings.

To detect changes in the population size, two approaches were executed. Neutrality tests, such as Tajima’s *D*^56^and Fu’s *Fs*^57^, were calculated in Arlequin. The historical dynamics of the population size and gene flow were profiled with skyline plots implemented in Migrate-n version 3.6.4^58^.

### Phylogenetic inference and molecular dating

Under the median-joining (MJ) model, the genealogical topologies of chlorotypes and ribotypes were constructed with NETWORK Version 4.6.1.2^59^. To estimate the time of diversification and divergence of the lineages, phylogenetic trees were reconstructed using a Bayesian approach and BEAST version 1.8.0^60^. The mutation rates were carefully selected as 4.8 × 10^−9^ s/s/y for nrITS^61^ and 1.7 × 10^−9^ s/s/y for cpDNA^62^, which were reciprocally calibrated according to the relative divergence (*Da*) of nrITS and cpDNA fragments within and among the genera investigated (Fig. 2a). In addition, based on the best-fit models (TPM3 + G and TVM for cpDNA and nrITS, respectively) estimated with jModelTest version 0.1.1^63^, the supportive index for each branch was also estimated using the Maximum Likelihood (ML, PhyML version 3.0)^64^ and Bayesian Inference (BI, MrBayes Version 3.2.1)^65^ methods (For detailed settings, refer to Yin *et al*.)^11^.

To further reconstruct the ancestral state and dispersal history of *A. squarrosum*, BEAST trees were also used in the Bayesian Binary Method (BBM) analysis implemented in RASP version 3.0^66^. Three *Corispermum* species were removed to prevent sampling bias. Ten MCMC chains were applied with one million generations under the JC + G model and sampled every 100 generations.

### Ecological niche modelling

Based on a total of 125 localities, including our own collection sites and records from the Chinese Virtual Herbarium (http://www.cvh.org.cn/), the present distribution range of *A. squarrosum* was predicted under maximum entropy modelling with MAXENT version 3.3.3 after 100 replicates of cross-validation with default settings^67^. Twelve independent environmental variables at a resolution of 2.5 arc-minutes^68^ from the WorldClim database version 1.4 (1950–2000, http://www.worldclim.org/) were used to model the niche of this species. In addition, a “maximum training sensitivity plus specificity” threshold was used to determine suitable/unsuitable habitat^69^. The goodness of each run was evaluated with the area under the receiving operator characteristics curve (AUC over 0.9)^70^,^71^. Subsequently, the current model was projected into the past (LGM, ~21 Kya and LIG, ~140 Kya) and future (the year 2070 with moderate carbon release based on the average between the year 2061 and 2080) layers to predict the potential distribution range of *A. squarrosum*. Because of its relatively good performance in prediction on the climate over East Asia^72^,^73^, the Model for Interdisciplinary Research on Climate (MIROC) at a resolution of 2.5 arc-minutes^74^ from WorldClim database version 1.4 was used to project all bioclimatic layers.

## Additional Information

**How to cite this article**: Qian, C. *et al*. Population dynamics of *Agriophyllum squarrosum*, a pioneer annual plant endemic to mobile sand dunes, in response to global climate change. *Sci. Rep*. **6**, 26613; doi: 10.1038/srep26613 (2016).

## Supplementary Material

Supplementary Information

## Figures and Tables

**Table 1:** Estimates of average gene diversity within populations (*H*_S_), total gene diversity (*H*_T_), inter-population differentiation (*G*st), and number of substitution types (*N*st) (mean ± SE in parentheses) for ribotype and chlorotype sequences.

	Grouping	H_S_	H_T_	Gst	Nst	Vs	Vt
nrITS	GuD	0.690(0.117)	0.854(0.063)	0.192(0.169)	0.178(0.184)	0.699(0.302)	0.850(0.251)
	Ta-KuD	0.473(0.052)	0.603(0.035)*	0.216(0.107)	0.142(0.066)	0.510(0.057)	0.595(0.049)^*^
	Central	0.559(0.051)	0.630(0.050)^*^	0.113(0.024)^*^	0.113(0.028)^*^	0.559(0.061)	0.630(0.065)
	QTP	0.679(0.077)	0.878(0.060)	0.227(0.058)	0.385(0.130)	0.561(0.080)	0.912(0.067)
	East	0.564(0.065)	0.595(0.071)	0.052(0.044)	−0.039(NC)	0.608(0.120)	0.586(0.117)
	Total	0.569(0.035)^*^	0.750(0.037)^*^	0.241(0.037)^*^	0.311(0.050)^*^	0.518(0.043)^*^	0.751(0.067)
cpDNA	GuD	0.278(0.278)	0.833(0.127)	0.667(0.398)	0.500(0.473)	0.395(0.395)	0.789(0.242)
	Ta-KuD	–	–	–	–	–	–
	Central	0.397(0.067)	0.772(0.034)^*^	0.486(0.086)	0.508(0.090)	0.380(0.075)	0.772(0.067)
	QTP	0.000(0.000)	0.500(0.250)	1.000(NC)	1.000(NC)	0.000(0.000)	0.500(0.250)
	East	0.133(0.133)	0.200(0.160)	0.333(NC)	0.333(NC)	0.133(0.133)	0.200(0.160)
	Total	0.283(0.052)	0.819(0.028)^*^	0.655(0.063)	0.742(0.074)	0.212(0.061)	0.820(0.121)

^*^Significance level P < 0.05

**Table 2:** Analysis of the molecular variance of nrITS and cpDNA for five groups.

Groups	Source of Variation	nrITS					cpDNA				
		d.f.	SS	VC	PV (%)	Fixation index	d.f.	SS	VC	PV(%)	Fixation index
GuD	Among populations	2	4.583	0.182 Va	17.780	F_ST_ = 0.178*	2	5.667	0.472 Va	33.330	F_ST_ = 0.333*
	Within populations	21	17.625	0.839 Vb	82.220		9	8.500	0.944 Vb	66.670	
	Total	23	22.208	1.021			11	14.167	1.417		
Ta-KuD	Among populations	4	5.646	0.099 Va	16.710	F_ST_ = 0.167	4	0.000	0.000 Va	0.000	F_ST_ = 0.000
	Within populations	43	21.125	0.491 Vb	83.290		19	0.000	0.000 Vb	0.000	
	Total	47	26.771	0.590			23	0.000	0.000		
Central	Among populations	28	30.534	0.069 Va	11.26	F_ST_ = 0.113*	28	40.603	0.294 Va	51.55	F_ST_ = 0.516*
	Within populations	203	109.875	0.541 Vb	88.74		87	24.000	0.276 Vb	48.45	
	Total	231	140.409	0.610			115	64.603	0.569		
QTP	Among populations	3	15.438	0.536 Va	38.480	F_ST_ = 0.385*	3	6.000	0.500 Va	100.000	F_ST_ = 1.000
	Within populations	28	24.000	0.857 Vb	61.520		12	0.000	0.000 Vb	0.000	
	Total	31	39.438	1.393			15	6.000	0.500		
East	Among populations	4	1.100	−0.015 Va	**−**3.900	F_ST_ = −0.039	4	0.800	0.033 Va	33.330	F_ST_ = 0.333
	Within populations	35	13.750	0.394 Vb	103.900		15	1.000	0.067 Vb	66.670	
	Total	39	14.850	0.378			19	1.800	0.100		
All 5 groups	Among groups	4	64.523	0.269 Va	28.75	F_CT_ = 0.288*	4	85.653	0.735 Va	59.71	F_CT_ = 0.597*
	Among populations within groups	41	57.301	0.102 Vb	10.94	F_SC_ = 0.154*	41	53.070	0.260 Vb	21.14	F_SC_ = 0.525*
	Within populations	330	186.375	0.565 Vc	60.31	F_ST_ = 0.397*	142	33.500	0.236 Vc	19.15	F_ST_ = 0.808*
	Total	375	308.199	0.936			187	172.223	1.232		

d.f., degrees of freedom; SS, sum of squares; VC, variance component; *F*_ST_, correlation within populations relative to total; *F*_SC_, correlation within populations relative to groups; *F*_CT_, correlation of haplotypes within groups relative to total; *P < 0.001 (10,000 permutations).

**Table 3:** Pairwise genetic differentiation (Fst) among 5 groups estimated from nrITS sequences (upper part) and cpDNA sequences (lower part) of *A. squarrosum*.

	GuD	Ta-KuD	QTP	Central	East
GuD	–	0.090**	0.131**	0.015	0.484***
Ta-KuD	0.419***	–	0.133***	0.120***	0.631***
QTP	0.360***	0.658***	–	0.200***	0.543***
Central	0.203***	0.133***	0.296***	–	0.516***
East	0.806***	0.979***	0.909***	0.797***	–

Significance level: *P < 0.05, **P < 0.01, ***P < 0.001; NS, not significant.

**Table 4:** Statistics for neutrality tests and mismatch distribution analysis for each group.

Regions	nrITS				cpDNA			
	Mismatch distribution		Neutrality tests		Mismatch distribution		Neutrality tests	
	*SSD* (*P*-value)	*RAG* (*P*-value)	Fu’s *Fs* (*P*-value)	Tajiam’s*D* (*P*-value)	*SSD* (*P*-value)	*RAG* (*P*-value)	Fu’s *Fs* (*P*-value)	Tajiam’s*D* (*P*-value)
GuD	0.006 (0.570)	0.037 (0.760)	−1.876 (0.135)	−0.555 (0.335)	0.153 (0.150)	0.524 (0.060)	1.563 (0.825)	−1.500 (0.058)
Ta-KuD	0.120 (0.160)	0.446 (0.080)	2.464 (0.892)	1.429 (0.916)	0.000 (0.000)	0.000 (0.000)	0.000 (N.A)	0.000 (1.000)
Central	0.008 (0.540)	0.053 (0.790)	−**21.019 (0.000)**	−**1.672 (0.017)**	0.024 (0.010)	0.143 (0.000)	−1.44 (0.264)	−0.914 (0.194)
QTP	0.012 (0.540)	0.034 (0.750)	−0.351 (0.464)	0.841 (0.831)	0.320 (0.000)	0.680 (0.900)	2.177 (0.841)	0.650 (0.819)
East	0.009 (0.230)	0.117 (0.170)	−2.127 (0.072)	−0.896 (0.200)	0.015 (0.290)	0.422 (0.460)	−0.097 (0.228)	−0.592 (0.252)
ALL	0.007 (0.450)	0.038 (0.720)	−**27.231 (0.000)**	−**1.801 (0.008)**	0.016 (0.000)	0.085 (0.020)	−2.234 (0.226)	−1.430 (0.051)

*SSD*, sum of squared deviation under expansion model; *RAG*, Harpending’s raggedness index; *D*, Tajima’s *D* test statistic; *F*s, Fu’s *F*s test statistic.
