# Supplementary material for: Population dynamics of Agriophyllum squarrosum, a pioneer annual plant endemic to mobile sand dunes, in response to global climate change
Source: Sci Rep. 2016 May 23;6:26613. doi: 10.1038/srep26613 (PMC4876407; doi:10.1038/srep26613)
Supplement: Supplementary Information [file srep26613-s1.doc]

**Supplementary Information**

**Population dynamics of *Agriophyllum squarrosum*, a pioneer annual plant endemic to mobile sand dunes, in response to global climate change**

Chaoju Qian, Hengxia Yin, Yong Shi, Jiecai Zhao, Chengliang Yin, Wanyin Luo, Zhibao Dong, Guoxiong Chen, Xia Yan, Xiao-Ru Wang and Xiao-Fei Ma


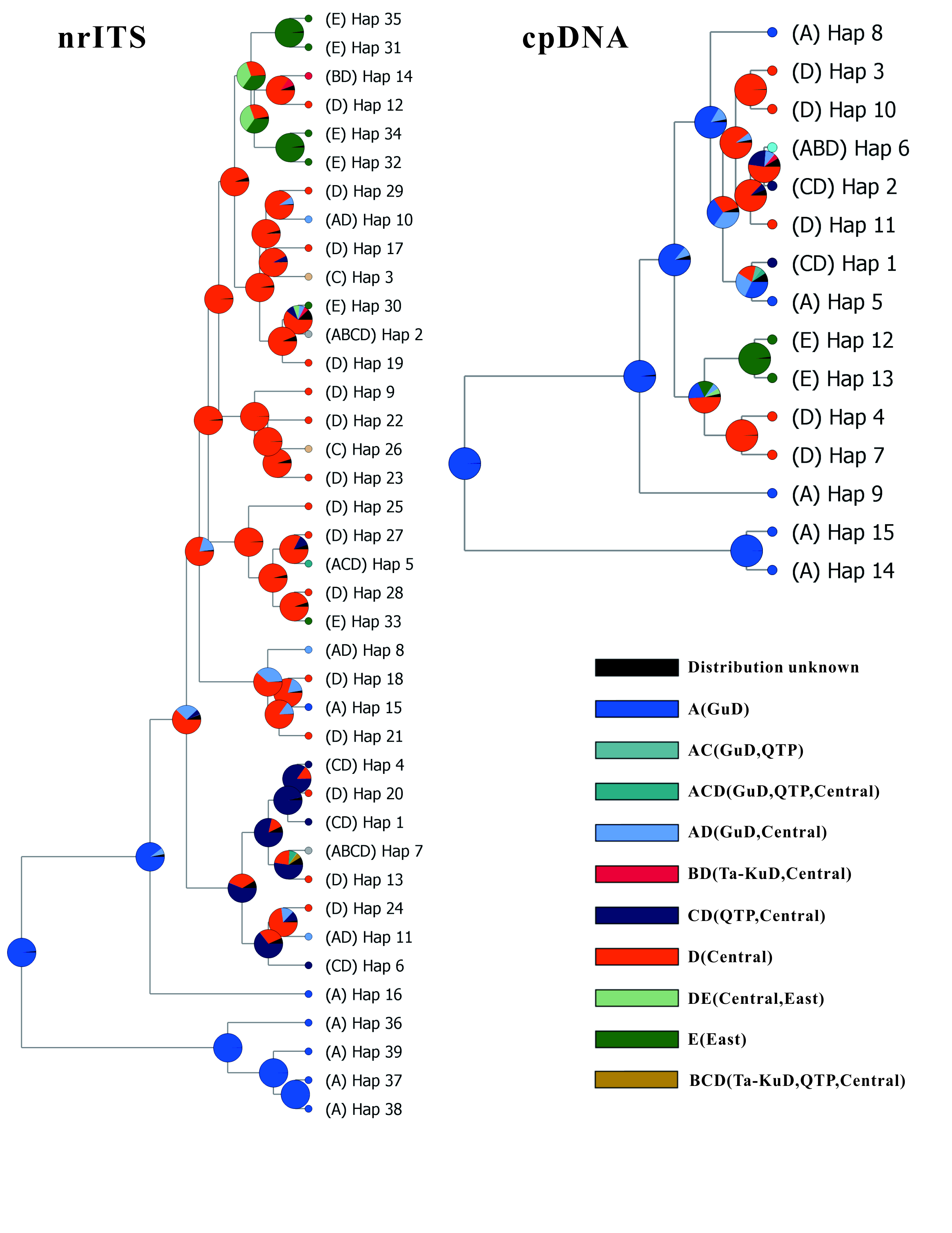


**Fig. S1** The results of Rasp for *A. squarrosum* based on nrITS data and cpDNA data, respectively, different color represent different origin group as shown in the lower right corner.


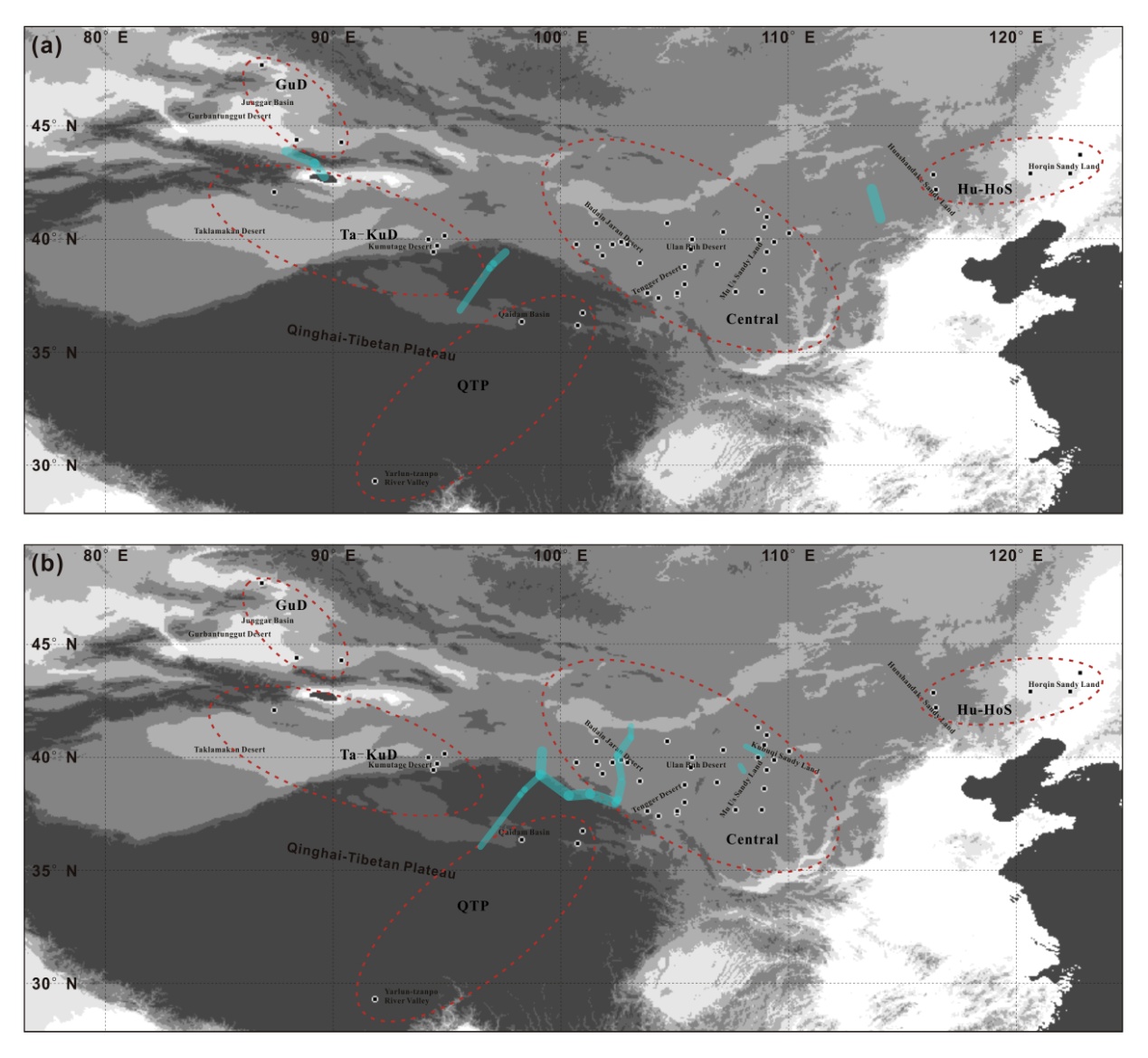


**Fig. S2** Population sampling and grouping of *A. squarrosum* and barriers. (a) for nrITS, (b) for cpDNA. The blue bold line represent the genetic barriers between populations, and different width of the barrier lines representing different bootstrap values. These figures originated from the software packages of Diva-GIS version 7.5.0 (<http://www.diva-gis.org/>), and then modified by Q,C.J. with CorelDraw X6 (Corel Corporation, Ottawa, Canada).

Table S1 Grouping and summary of genetic diversity of each population in *A. squarrosum* for ribotypes

| Grouping | Locations | Haplotypes(Individuals)* | s | h | Hd | Pi |
| --- | --- | --- | --- | --- | --- | --- |
| **GuD** | BEJX | R2(2)R7(2)R15(2)R16(2) | 6 | 4 | 0.857 | 0.0044 |
|  | FK | R2(6)R5(1)R8(1) | 2 | 3 | 0.464 | 0.0009 |
|  | ML | R2(1)R7(4)R10(2)R11(1) | 3 | 4 | 0.750 | 0.0026 |
|  | **Total** |  | 8 | 8 | 0.804 | 0.0030 |
| **Ta-KuD** | YG | R2(6)R7(2) | 2 | 2 | 0.429 | 0.0015 |
|  | DH | R2(6)R7(2) | 2 | 2 | 0.429 | 0.0015 |
|  | SSG | R7(6)R14(2) | 3 | 2 | 0.429 | 0.0022 |
|  | AKS | R2(4)R7(3)R14(1) | 3 | 3 | 0.679 | 0.0023 |
|  | JSJ | R2(4)R7(12) | 2 | 2 | 0.400 | 0.0014 |
|  | **Total** |  | 3 | 3 | 0.563 | 0.0020 |
| **QTP** | YJ | R2(2)R5(3)R26(3) | 2 | 3 | 0.750 | 0.0018 |
|  | DL | R1(4)R2(1)R7(3) | 4 | 3 | 0.679 | 0.0028 |
|  | TGX | R1(6)R2(1)R3(1) | 6 | 3 | 0.464 | 0.0038 |
|  | QHH | R2(3)R4(2)R5(2)R6(1) | 4 | 4 | 0.821 | 0.0033 |
|  | **Total** |  | 8 | 8 | 0.833 | 0.0044 |
| **Central** | M4 | R2(2)R4(2)R7(2)R21(1)R22(1) | 5 | 5 | 0.893 | 0.0035 |
|  | S136 | R1(2)R2(2)R7(2)R23(1)R24(1) | 4 | 5 | 0.893 | 0.0035 |
|  | M6 | R2(6)R5(1)R11(1) | 2 | 3 | 0.464 | 0.0009 |
|  | M1 | R2(1)R7(4)R10(2)R20(1) | 5 | 4 | 0.750 | 0.0034 |
|  | M5 | R2(3)R4(1)R7(2)R10(1)R19(1) | 5 | 5 | 0.857 | 0.0031 |
|  | AYQ | R2(2)R7(3)R8(1)R10(1)R11(1) | 4 | 5 | 0.857 | 0.0028 |
|  | WLJ | R2(4)R7(3)R14(1) | 3 | 3 | 0.679 | 0.0023 |
|  | M2 | R2(4)R6(1)R7(1)R10(1)R11(1) | 4 | 5 | 0.786 | 0.0022 |
|  | MQ | R2(4)R5(1)R7(3) | 3 | 3 | 0.679 | 0.0023 |
|  | XJ | R2(7)R4(1) | 3 | 2 | 0.250 | 0.0013 |
|  | JT | R2(3)R4(2)R7(1)R12(2) | 4 | 4 | 0.821 | 0.0033 |
|  | SPT | R2(7)R17(1) | 1 | 2 | 0.250 | 0.0004 |
|  | TGL | R2(8) | 0 | 1 | 0.000 | 0.0000 |
|  | LJT | R2(6)R7(1)R11(1) | 2 | 3 | 0.464 | 0.0012 |
|  | YLH | R2(5)R8(2)R18(1) | 2 | 3 | 0.607 | 0.0013 |
|  | JCHK | R2(4)R7(4) | 2 | 2 | 0.571 | 0.0020 |
|  | JLT | R2(6)R4(2) | 3 | 2 | 0.429 | 0.0022 |
|  | BYWS | R2(7)R7(1) | 2 | 2 | 0.250 | 0.0009 |
|  | BLG | R2(6)R7(2) | 2 | 2 | 0.429 | 0.0015 |
|  | DLSH | R2(3)R7(1)R9(1)R12(1)R13(1)R14(1) | 5 | 6 | 0.893 | 0.0031 |
|  | DSHT | R2(7)R7(1) | 2 | 2 | 0.250 | 0.0009 |
|  | HJNE | R2(6)R7(2) | 2 | 2 | 0.429 | 0.0015 |
|  | XSW | R2(8) | 0 | 1 | 0.000 | 0.0000 |
|  | HJQ | R2(3)R7(3)R27(1)R28(1) | 5 | 4 | 0.786 | 0.0034 |
|  | KY | R2(3)R5(1)R7(1)R9(3) | 4 | 4 | 0.786 | 0.0022 |
|  | WSQ | R2(7)R25(1) | 2 | 2 | 0.250 | 0.0009 |
|  | MMH | R2(2)R5(3)R11(1)R19(1)R29(1) | 4 | 5 | 0.857 | 0.0022 |
|  | JB | R2(4)R5(4) | 1 | 2 | 0.571 | 0.0010 |
|  | BB | R2(6)R5(1)R10(1) | 2 | 3 | 0.464 | 0.0010 |
|  | **Total** |  | 20 | 25 | 0.628 | 0.0021 |
| **Hu-HoS** | HLHR | R30(5)R31(3) | 1 | 2 | 0.536 | 0.0009 |
|  | DLN | R30(6)R35(2) | 2 | 2 | 0.429 | 0.0015 |
|  | NM | R30(4)R31(3)R32(1) | 2 | 3 | 0.679 | 0.0013 |
|  | AEX | R30(6)R31(2) | 1 | 2 | 0.429 | 00007 |
|  | DALIN | R30(2)R31(4)R33(1)R34(1) | 4 | 4 | 0.750 | 0.0023 |
|  | **Total** |  | 5 | 6 | 0.590 | 0.0013 |
| **All 5 groups** | | - | 29 | 33 | 0.691 | 0.0025 |

*including the indels

Table S2 Grouping and summary of genetic diversity of each population in *A. squarrosum* for chlorotypes

| Grouping | Locations | Haplotypes(Individuals)* | s | h | Hd | Pi |
| --- | --- | --- | --- | --- | --- | --- |
| **GuD** | BEJX | C5(2)C8(1)C9(1) | 9 | 3 | 0.833 | 0.0014 |
|  | FK | C5(4) | 0 | 1 | 0.000 | 0.0000 |
|  | ML | C6(4) | 0 | 1 | 0.000 | 0.0000 |
|  | **Total** |  | 9 | 4 | 0.682 | 0.0006 |
| **Ta-KuD** | YG | C6(4) | 0 | 1 | 0.000 | 0.0000 |
|  | DH | C6(4) | 0 | 1 | 0.000 | 0.0000 |
|  | SSG | C6(4) | 0 | 1 | 0.000 | 0.0000 |
|  | AKS | C6(4) | 0 | 1 | 0.000 | 0.0000 |
|  | JST | C6(8) | 0 | 1 | 0.000 | 0.0000 |
|  | **Total** |  | 0 | 1 | 0.000 | 0.0000 |
| **QTP** | YJ | C2(4) | 0 | 1 | 0.000 | 0.0000 |
|  | DL | C1(4) | 0 | 1 | 0.000 | 0.0000 |
|  | TGX | C1(4) | 0 | 1 | 0.000 | 0.0000 |
|  | QHH | C1(4) | 0 | 1 | 0.000 | 0.0000 |
|  | **Total** |  | 1 | 2 | 0.400 | 0.0001 |
| **Central** | M4 | C2(4) | 0 | 1 | 0.000 | 0.0000 |
|  | S136 | C2(2)C6(2) | 0 | 1 | 0.000 | 0.0000 |
|  | M6 | C2(4) | 0 | 1 | 0.000 | 0.0000 |
|  | M1 | C6(4) | 0 | 1 | 0.000 | 0.0000 |
|  | M5 | C2(3)C6(1) | 0 | 1 | 0.000 | 0.0000 |
|  | AYQ | C1(2)C6(2) | 1 | 2 | 0.667 | 0.0002 |
|  | WLJ | C2(4) | 0 | 1 | 0.000 | 0.0000 |
|  | M2 | C2(2)C6(1)C10(1) | 1 | 2 | 0.500 | 0.0002 |
|  | MQ | C1(4) | 0 | 1 | 0.000 | 0.0000 |
|  | XJ | C1(3)C6(1) | 1 | 2 | 0.500 | 0.0002 |
|  | JT | C6(4) | 0 | 1 | 0.000 | 0.0000 |
|  | SPT | C6(4) | 0 | 1 | 0.000 | 0.0000 |
|  | TGL | C6(4) | 0 | 1 | 0.000 | 0.0000 |
|  | LJT | C2(1)C4(1)C6(2) | 1 | 2 | 0.500 | 0.0002 |
|  | YLH | C2(3)C6(1) | 0 | 1 | 0.000 | 0.0000 |
|  | JCHK | C2(4) | 0 | 1 | 0.000 | 0.0000 |
|  | JLT | C2(4) | 0 | 1 | 0.000 | 0.0000 |
|  | BYWS | C2(3)C6(1) | 0 | 1 | 0.000 | 0.0000 |
|  | BLG | C2(2)C4(2) | 1 | 2 | 0.667 | 0.0002 |
|  | DLSH | C3(4) | 0 | 1 | 0.000 | 0.0000 |
|  | DSHT | C3(4) | 0 | 1 | 0.000 | 0.0000 |
|  | HJNE | C2(2)C3(1)C4(1) | 2 | 3 | 0.833 | 0.0003 |
|  | XSW | C2(1)C3(1)C4(1)C7(1) | 3 | 4 | 1.000 | 0.0005 |
|  | HJQ | C4(2)C6(2) | 1 | 2 | 0.667 | 0.0002 |
|  | KY | C2(3)C4(1) | 1 | 2 | 0.500 | 0.0002 |
|  | WSQ | C4(3)C6(1) | 1 | 2 | 0.500 | 0.0002 |
|  | MMH | C3(3)C4(1) | 2 | 2 | 0.500 | 0.0003 |
|  | JB | C4(1)C6(2)C11(1) | 2 | 3 | 0.833 | 0.0003 |
|  | BB | C4(1)C6(1)C11(2) | 2 | 3 | 0.833 | 0.0003 |
|  | **Total** |  | 6 | 7 | 0.553 | 0.0002 |
| **Hu-HoS** | HLHR | C12(4) | 0 | 1 | 0.000 | 0.0000 |
|  | DLN | C12(2)C13(2) | 1 | 2 | 0.667 | 0.0002 |
|  | NM | C12(4) | 0 | 1 | 0.000 | 0.0000 |
|  | AEX | C12(4) | 0 | 1 | 0.000 | 0.0000 |
|  | DALIN | C12(4) | 0 | 1 | 0.000 | 0.0000 |
|  | **Total** |  | 1 | 2 | 0.189 | 0.0001 |
| **All 5 groups** | | - | 18 | 12 | 0.646 | 0.0004 |

*including the indels

**Table S3** List of the bioclimatic variables used to develop the ecological niche models and the variable contributions.

| **Variable** | **Percent contribution** | **Permutation importance** | **Discription** |
| --- | --- | --- | --- |
| **bio_19** | **40.9** | **49.9** | **Precipitation of Coldest Quarter** |
| **bio_11** | **18.4** | **13.3** | **Mean Temperature of Coldest Quarter** |
| bio_9 | 8.6 | 0.4 | Mean Temperature of Driest Quarter |
| bio_18 | 7.5 | 6.8 | Precipitation of Warmest Quarter |
| bio_5 | 7.2 | 2.5 | Max Temperature of Warmest Month |
| Bio_8 | 6.6 | 0.9 | Mean Temperature of Wettest Quarter |
| bio_4 | 4.3 | 0.9 | Temperature Seasonality  (standard deviation *100) |
| bio_13 | 2.6 | 6.3 | Precipitation of Wettest Month |
| bio_2 | 2.6 | 7.5 | Mean Diurnal Range  (Mean of monthly (max- min temp)) |
| bio_15 | 1 | 8.2 | Precipitation Seasonality  (Coefficient of Variation) |
| bio_3 | 0.2 | 1.1 | Isothermality (BIO2/BIO7) (* 100) |
| bio_14 | 0.2 | 2.2 | Precipitation of Driest Month |

**Table S4** Details of the locality information of the sampled populations.

| **No.** | **group** | **Population code** | **Location**  **(All in China)** | **Latitude**  **(N°)** | **Longitude**  **(E°)** | **Altitude**  **(m)** |
| --- | --- | --- | --- | --- | --- | --- |
| **1** | GuD | BEJX | Buerjinxi, Gurbantunggut | 47°39′26.48″ | 86°36′20.82″ | 971 |
| **2** | GuD | FK | Fukang, Gurbantunggut | 44°22′17.48″ | 88°8′31.20″ | 460 |
| **3** | GuD | ML | Mulei, Gurbantunggut | 44°14′25.93″ | 90°8′33.84″ | 741 |
| **4** | Ta-KuD | YG | Yangguan, Kumtag | 39°57′37.80″ | 93°59′56.34″ | 1223 |
| **5** | Ta-KuD | DH | Dunhuang,Kumtag | 40°6′32.61″ | 94°40′3.68″ | 1138 |
| **6** | Ta-KuD | SSG | Shashangou, Kumtag | 39°39′51.46″ | 94°22′0.33″ | 1631 |
| **7** | Ta-KuD | AKS | Akesai, Kumtag | 39°25′14.52″ | 94°12′36.76″ | 2539 |
| **8** | Ta-KuD | JST | Jinshatan, Takalamakan | 42°2′55.32″ | 87°9′41.58″ | 1033 |
| **9** | QTP | YJ | Yajiang,Yarlun-tzanpoRiver Valley | 29°14′58.04″ | 91°35′53.92″ | 3844 |
| **10** | QTP | DL | Dulan, Qaidam | 36°25′25.77″ | 98°7′25.35″ | 3123 |
| **11** | QTP | TGX | Tiegaixiang, Qaidam | 36°10′02.09″ | 100°34′13.85″ | 2905 |
| **12** | QTP | QHH | Qinghaihu, Qaidam | 36°43′3.13″ | 100°47′17.46″ | 3251 |
| **13** | Central | M4 | M4, Badan Jaran | 40°38′12.46″ | 101°25′52.09″ | 1110 |
| **14** | Central | S136 | S136, Badan Jaran | 39°43′49.14″ | 100°33′19.35″ | 1369 |
| **15** | Central | M6 | M6, Badan Jaran | 39°45′6.92″ | 102°9′28.38″ | 1234 |
| **16** | Central | M1 | M1, Badan Jaran | 39°38′8.81″ | 101°29′10.37″ | 1407 |
| **17** | Central | M5 | M5, Badan Jaran | 39°49′10.80″ | 102°34′14.20″ | 1452 |
| **18** | Central | AYQ | Ayouqi,Badan Jaran | 39°15′6.45″ | 101°40′52.02″ | 1523 |
| **19** | Central | WLJ | Wuliji,Badan Jaran | 40°38′45.34″ | 104°33′36.49″ | 1253 |
| **20** | Central | M2 | M2, Badan Jaran | 39°43′35.01″ | 102°47′38.70″ | 1559 |
| **21** | Central | MQ | Minqin | 38°53′35.02″ | 103°20′18.56″ | 1334 |
| **22** | Central | XJ | Xinjin,Tengger | 37°32′45.45″ | 103°41′37.13″ | 1789 |
| **23** | Central | JT | Jingtai, Tengger | 37°22′0.80″ | 104°8′47.92″ | 1606 |
| **24** | Central | SPT | Shapotou,Tengger | 37°32′33.58″ | 105°02′10.14″ | 1656 |
| **25** | Central | TGL | Tengger , Tengger | 37°34′26.82″ | 105°1′15.97″ | 1276 |
| **26** | Central | LJT | Luanjintan,Tengger | 37°56′51.80″ | 105°19′39.59″ | 1321 |
| **27** | Central | YLH | Yuelianghu,Tengger | 38°44′45.83″ | 105°21′19.76″ | 1286 |
| **28** | Central | JCHK | Jichakou, Ulan Buh | 39°31′12.24″ | 105°35′52.23″ | 1063 |
| **29** | Central | JLT | Jilantai, Ulan Buh | 39°55′40.01″ | 105°40′34.77″ | 1021 |
| **30** | Central | BYWS | Bayinwusu, Ulan Buh | 39°56′31.40″ | 108°34′46.59″ | 1266 |
| **31** | Central | BLG | Balagong,Kubuqi | 40°15′42.68″ | 107°2′6.34″ | 1072 |
| **32** | Central | DLSH | Delingshan,Kubuqi | 41°16′24.08″ | 108°35′23.56″ | 1048 |
| **33** | Central | DSHT | Dashetai, Kubuqi | 40°56′18.00″ | 108°58′53.15″ | 1020 |
| **34** | Central | HJNE | Hangjinnaoer,Kubuqi | 40°29′9.21″ | 108°51′32.04″ | 1045 |
| **35** | Central | XSW | Xiangshawa,Kubuqi | 40°14′30.32″ | 109°57′30.68″ | 1219 |
| **36** | Central | HJQ | Hangjinqi,Kubuqi | 39°51′0.97″ | 109°17′0.81″ | 1395 |
| **37** | Central | KY | Keyan,Mu Us | 39°24′28.45″ | 108°57′58.90″ | 1371 |
| **38** | Central | WSQ | Wushenqi, Mu Us | 38°33′23.36″ | 108°51′6.05″ | 1279 |
| **39** | Central | MMH | Miaomiaohu, Mu Us | 38°50′30.30″ | 106°49′34.02″ | 1270 |
| **40** | Central | JB | Jingbian, Mu Us | 37°37′20.05″ | 108°44′16.33″ | 1336 |
| **41** | Central | BB | Baobian, Mu Us | 37°36′50.84″ | 107°36′14.19″ | 1354 |
| **42** | East | HLHR | Haolaihure,Otindag | 42°50′15.30″ | 116°21′25.00″ | 1345 |
| **43** | East | DLN | Duolunna,Otindag | 42°10′06.70″ | 116°28′34.30″ | 1252 |
| **44** | East | NM | Naiman,Horqin | 42°52′29.68″ | 120°38′46.77″ | 373 |
| **45** | East | AEX | Aerxiang,Horqin | 42°52′4.80″ | 122°25′40.14″ | 251 |
| **46** | East | DALIN | Dalin, Horqin | 43°42′30.60″ | 122°50′36.30″ | 152 |

**Table S5** Twelve most variable regions in chloroplast genomes and one nrITS region detected in this study.

| **Code** | Region | primer（5’-3’） | | Sequence length(bp) | Tm (℃) | Reference |
| --- | --- | --- | --- | --- | --- | --- |
| **1** | *petB-petD* | *petB* | CAATCCACTTTGACTCGTTTT | 769 | 57 | Dong *et al*.(2012) |
|  |  | *petD* | GGTTCACCAATCATTGATGGTTC |  |  |  |
| **2** | *trnS^(GCU)^-*  *trnG^(UCC)^* | *trnS1* | AACGGATTAGCAATCCGACGCTTTA | 638 | 57 | Dong *et al*.(2012) |
|  |  | *trnG1* | CTTTTACCACTAAACTATACCCGC |  |  |  |
| **3** | *rbcL* | *rbcL-F* | TAGCTGCTGCTTGTGAGGTATGGA | 687 | 55 | Dong *et al*.(2012) |
|  |  | *rbcL-AR* | TGAGCCAACGAAGTATTTGC |  |  |  |
| **4** | *ndhc-trnV* | *Qndhc* | ATATCGAAACTCATTGCCCAT | 643 | 66 | This study |
|  |  | *QtrnV* | GTTCGAGTCCGTATAGCCCTA |  |  |  |
| **5** | *rpoB-trnC* | *rpoB* | ACAAAATCCTTCAAATTGTATCTGA | 664 | 53 | Dong *et al*.(2012) |
|  |  | *trnC* | TTTGTTAATCAGGCGACACCCGG |  |  |  |
| **6** | *ITS1-ITS4* | *ITS1* | TCCGTAGGTGAACCTGCGG | 586 | 60 | White *et al*.(1990) |
|  |  | *ITS4* | TCCTCCGCTTATTGATATGC |  |  | Baum *et al*.(1998) |
| **7** | *rps4* | *rps4-F* | ATGTCCCGTTATCGAGGACCT | 730 | 52 | Dong *et al*.(2012) |
|  |  | *rps4-R* | TACCGAGGGTTCGAATC |  |  |  |
| **8** | *trnS-trnG* | *trnS* | GCCgCTTTAGTCCACTCAGC | 800 | 57 | Dong *et al*.(2012) |
|  |  | *trnG* | GAACGAATCACACTTTTACCAC |  |  |  |
| **9** | *rpl32-trnL* | *rpl32-F* | GCGTATTCGTAAAAATATTTGGAA | 870 | 53 | Dong *et al*.(2012) |
|  |  | *trnL-R* | TTCCTAAGAGCAGCGTGTCTACC |  |  |  |
| **10** | *clpP* | *clpP-F* | GCTTGGGCTTCTCTTGCTGACA | 1030 | 64 | Dong *et al*.(2012) |
|  |  | *clpP-R* | TCCTAATCAACCGACTTTATCGAG |  |  |  |
| **11** | *trnT-psbD* | *trnT* | GCCCTTTTAACTCAGTGGTAGAG | 1010 | 57 | Dong *et al*.(2012) |
|  |  | *psbD* | CCAAATAGGAACTGGCCAATC |  |  |  |
| **12** | *trnL-trnF* | *trnL* | GGTTCAAGTCCCTCTATCCC | 410 | 55 | Dong *et al*.(2012) |
|  |  | *trnF* | ATTTGAACTGGTGACACGAG |  |  |  |
| **13** | *trnH-psbA* | *trnH* | CGCGCATGGTGGATTCACAAATC | 680 | 60 | Dong *et al*.(2012) |
|  |  | *psbA* | TGCATGGTTCCTTGGTAACTTC |  |  |  |

REFERENCES:

Baum, D.A., Small, R.L. & Wendel, J.F.(1998) Biogeography and floral evolution of baobabs (*Adansonia*, Bombacaceae) as inferred from multiple data sets. *Systematic Biology*, **47**,181-207.

Dong, W., Liu, J., Yu, J., Wang, L. & Zhou, S. (2012) Highly Variable Chloroplast Markers for Evaluating Plant Phylogeny at Low Taxonomic Levels and for DNA Barcoding. PLoS ONE, 7, e35071.

White, T.J., Bruns, T., Lee, S. & Taylor, J. (1990) *Amplification and direct sequencing of fungal ribosomal RNA genes for phylogenetics*. In: PCR Protocols: a guide to methods and applications. Academic Press, New York.
